# Peak limitation – revised definition of the peak limitation categories: MAK Value Documentation, addendum – Translation of the German version from 2024

**DOI:** 10.34865/mbpeakexpgte10_1ad

**Published:** 2025-03-31

**Authors:** Andrea Hartwig, MAK Commission

**Affiliations:** 1 Institute of Applied Biosciences. Department of Food Chemistry and Toxicology. Karlsruhe Institute of Technology (KIT) Adenauerring 20a, Building 50.41 76131 Karlsruhe Germany; 2 Permanent Senate Commission for the Investigation of Health Hazards of Chemical Compounds in the Work Area. Deutsche Forschungsgemeinschaft, Kennedyallee 40, 53175 Bonn, Germany. Further information: Permanent Senate Commission for the Investigation of Health Hazards of Chemical Compounds in the Work Area | DFG

**Keywords:** peak limitation, definition, category

## Abstract

The German Senate Commission for the Investigation of Health Hazards of Chemical Compounds in the Work Area (MAK Commission) revised the definition of the peak limitation categories to emphasize that the time required for an effect to develop is decisive for the categorization.

## Revised definition

The previous definition (Hartwig and MAK Commission 2017) distinguished between irritants (Category I) and substances with systemic effects (Category II). It was implied that substances in Category I have rapid effects while those in Category II require a certain amount of time to have an effect as they must first be absorbed. The time required for effects to develop is decisive; to make this clear, the categories have been re-defined (Table 1). The uncertainty whether substances which affect the lungs as the target organ should be classified as systemic agents, although they in fact have local effects, is thus avoided. If the effect is manifest only after a longer period of time, Category II applies. Also in the past, substances were classified in this way. Substances which have sensitizing effects on the airways were classified in Category I, as the peak concentrations are decisive for their effects. Because the MAK value does not offer protection against the induction and elicitation of sensitization, or only does so to a certain extent, such peaks must be limited. This is guaranteed by an excursion factor of 1, which Category II does not have.

**Tab. 1:** Excursion factors, duration, number per shift and interval between the peaks

**Category**	**Excursion factor**	**Duration**	**Number per shift**	**Minimum interval^c)^**
I Substances with immediate effects (irritants) or which cause sensitization of the airways	1^a)^	15 minutes, average value^b)^	4	1 hour
II Substances with delayed effects (systemic effects or effects in the lungs after repeated exposure)	2^a)^	15 minutes, average value	4	1 hour

^a)^ default value, or a substance-specific value (maximum 8)

^b)^ In certain cases, a momentary value (concentration which should not be exceeded at any time) can also be established.

^c)^ only for excursion factors > 1

## References

[ref_HBKW6YRD] Hartwig A., MAK Commission (2017). Peak limitation: Limitation of exposure peaks and short-term exposures. MAK Value Documentation, 2011. MAK Collect Occup Health Saf.

